# A Review of the Recent Developments in the Bioproduction of Polylactic Acid and Its Precursors Optically Pure Lactic Acids

**DOI:** 10.3390/molecules26216446

**Published:** 2021-10-26

**Authors:** Shiyong Huang, Yanfen Xue, Bo Yu, Limin Wang, Cheng Zhou, Yanhe Ma

**Affiliations:** 1 State Key Laboratory of Microbial Resources, Institute of Microbiology, Chinese Academy of Sciences, Beijing 100101, China; huangshiyong18@mails.ucas.ac.cn (S.H.); xueyf@im.ac.cn (Y.X.); mayanhe@im.ac.cn (Y.M.); 2 University of Chinese Academy of Sciences, Beijing 100049, China; 3 CAS Key Laboratory of Microbial Physiological and Metabolic Engineering, State Key Laboratory of Mycology, Institute of Microbiology, Chinese Academy of Sciences, Beijing 100101, China; yub@im.ac.cn

**Keywords:** lactic acid, polylactic acid, microbial production, renewable resource, clean fermentation, metabolic engineering

## Abstract

Lactic acid (LA) is an important organic acid with broad industrial applications. Considered as an environmentally friendly alternative to petroleum-based plastic with a wide range of applications, polylactic acid has generated a great deal of interest and therefore the demand for optically pure l- or d-lactic acid has increased accordingly. Microbial fermentation is the industrial route for LA production. LA bacteria and certain genetic engineering bacteria are widely used for LA production. Although some fungi, such as *Saccharomyces cerevisiae*, are not natural LA producers, they have recently received increased attention for LA production because of their acid tolerance. The main challenge for LA bioproduction is the high cost of substrates. The development of LA production from cost-effective biomasses is a potential solution to reduce the cost of LA production. This review examined and discussed recent progress in optically pure l-lactic acid and optically pure d-lactic acid fermentation. The utilization of inexpensive substrates is also focused on. Additionally, for PLA production, a complete biological process by one-step fermentation from renewable resources is also currently being developed by metabolically engineered bacteria. We also summarize the strategies and procedures for metabolically engineering microorganisms producing PLA. In addition, there exists some challenges to efficiently produce PLA, therefore strategies to overcome these challenges through metabolic engineering combined with enzyme engineering are also discussed.

## 1. Introduction

**Figure 1:**
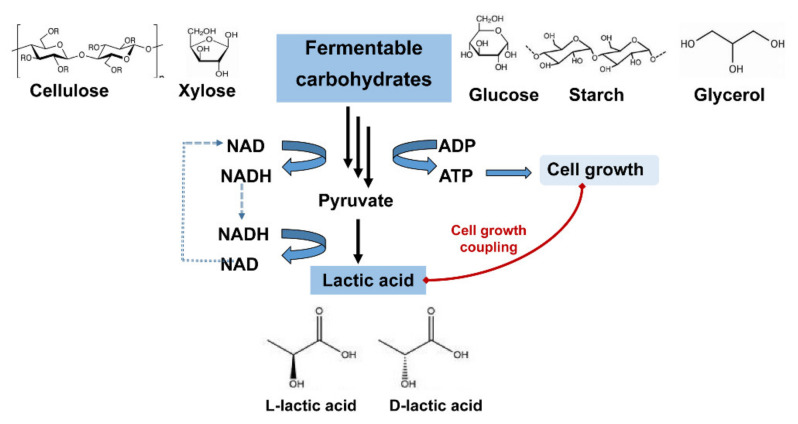
LA exists in two enantiomeric forms of l-lactic acid and d-lactic acid.

Lactic acid (CH_3_-CHOHCOOH, LA) is one of the most important building-block chemicals in the world. It contains a hydroxyl group and a carboxyl group. Because of its functional groups, LA could be used as a starting material for the synthesis of various chemicals, such as acrylic acid, 1,2-propanediol, 2,3-pentanedione [1]. The most widely applied use for LA is in the production of biodegradable polymers, polylactic acid (PLA) [2]. PLA is a biodegradable polymer, which has recently increased in global demand due to its increasing application as a bioplastic. Global plastics production totalled 368 million metric tons in 2020. At an estimated 10% replacement of fossil-fuel-based plastics, the overall demand for PLA is envisioned to reach 30 million tons per year. As the precursor of PLA, the demand for LA is estimated to reach 1960 kilotons by 2025 [3]. There are three forms of LA: l-lactic acid, d-lactic acid and racemic mixtures (dl-lactic acid) [4]. LA exists in two enantiomeric forms of l-lactic acid and d-lactic acid (Figure 1), and optically pure l-lactic acid and optically pure d-lactic acid are considered to have more value than racemic mixtures. l-lactic acid is widely used in the food industry. Optically pure l-lactic acid (≥99%) is the main precursor of PLA, and the addition of optically pure d-lactic acid may change the mechanical properties of PLA. Therefore, two isomers of LA have recently received increasing focus [5]. LA can be generated either by chemical synthesis from hydrocarbon-based sources or by microbial fermentation routes. A racemic form of d-/l-lactic acid is obtained through chemical routes, which have limited applications, whereas optical pure d- or l-lactic acid can be obtained by microbial fermentation [6]. Microbial production of LA has more benefits, including cheap raw materials and mild production conditions. At present, microbial fermentation is the main industrial route for LA production [3].

**Figure 2:**
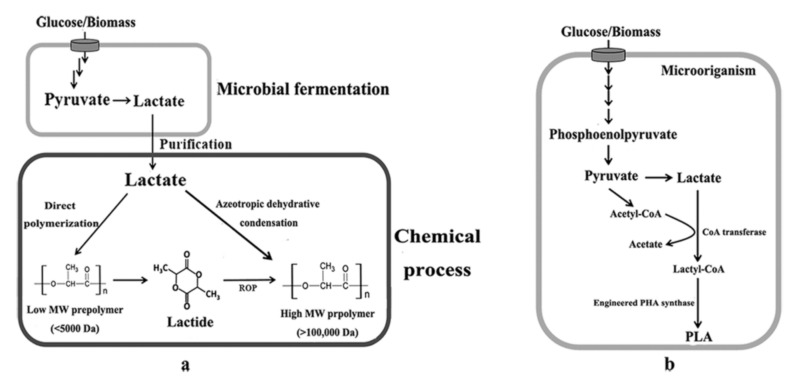
The process of PLA synthesis. (**a**) bio-chemical hybrid process; (**b**) one-step fermentative production by metabolically engineered microorganisms.

On the other hand, as the most widely applied use of LA and one of the most promising biodegradable plastics, PLA possesses much more outstanding advantages compared to other polymers, since it has biodegradability, biocompatibility, clarity, and superior barrier properties, along with suitable material properties for general performance plastics [7,8,9]. PLA is traditionally synthesized by bio-chemical hybrid process (Figure 2a), in which optical pure l-lactic acid or d-lactic acid as the monomer of PLA firstly is produced by microbial fermentation from renewable resources such as wheat, straw, corn, and sorghum [10], and then PLA is synthesized via the ring opening polymerization (ROP) of lactide, a cyclic dimer of lactate, or by the direct solvent-based azeotropic dehydrative condensation of LA [11,12,13]. PLA synthesis process usually requires catalysts under rigorously controlled conditions (temperature, pressure, and pH) and long polymerization times, which leads to high energy consumption [14]. In addition, the toxicity caused by the use of metal catalysts seriously affects the applications of PLA as biomedical and food-packaging materials [11]. As an alternative to the traditional production process, one-step fermentative production of PLA has recently been developed by employing metabolically engineered microorganisms (Figure 2b), such as *Escherichia coli* [15,16,17,18,19], *Sinorhizobium meliloti* and *Pseudomonas putida* [20].

Although there are some reviews on LA production, few reviews focus on the production of optically pure LA, which is the precursor for biodegradable plasmics, PLA. LA production will increase significantly over the coming years, mainly to provide PLA manufactures. A racemic mixture of dl-lactic acid is the common product for most LA-producing strains. dl-lactic acid cannot be utilized in specific industrial applications where only optically pure LA is desired. In this paper, we have gathered our accumulated knowledge on the main achievements in LA bio-production, especially in optically pure l-lactic acid and optically pure d-lactic acid fermentation. In addition, the detailed procedure and the latest research progresses of one-step fermentative production of PLA, including the exploration of key enzymes for polymerization of lactate, designing of microorganism chassis cell by metabolic engineering are also reviewed and discussed.

## 2. l-Lactic Acid Biosynthesis

Many microorganisms have been reported to have the ability to produce l-lactic acid, such as fungi, *Lactobacillus* species, *Bacillus coagulans*, and various genetically modified strains. LA is produced from carbohydrates in a microorganism. There are two pathways for LA production: homolactic fermentation and heterolactic fermentation. In heterolactic fermentation, ethanol, acetic acid and CO_2_ are formed in addition to LA as the end product. The theoretical yield is only 0.5 g LA/g glucose [2]. In homolactic fermentation, LA is produced from carbohydrates with the regeneration of NADH (Figure 1). Pyruvate produced by the glycolytic breakdown of carbohydrates is converted into l-lactic acid and d-lactic acid by NAD-dependent l-lactate dehydrogenanse (EC 1.1.1.27) and NAD-dependent d-lactate dehydrogenanse (EC 1.1.1.28), respectively [21]. For homofermentative microorganisms, LA production is growth coupling. LA is obtained as the sole product in homolactic fermentation. Therefore, most industrial LA-producing strains are homofermentative microorganisms.

### 2.1. l-Lactic Acid Producing Strains

l-lactic acid can be produced by several microorganisms classified into bacteria, fungi, cyanobacteria, and algae (Table 1). The filamentous fungus *Rhizopus oryzae* is a natural l-lactic acid producer. The amylolytic characteristic enables *R. oryzae* to utilize starchy biomasses without prior saccharification. A concentration of 162 g/L l-lactic acid, with a productivity of 6.23 g/L·h, was obtained using fed-batch strategy [22]. Different renewable resources, including molasses, raw starch materials, and lignocellulosic biomass, have been reported to be used to produce l-lactic acid using *Rhizopus* strains. However, low conversion rate and undesirable by-products production limits the industrial uses of *Rhizopus* strains [23]. During LA production, the end-product LA lowers the pH of the medium, and thus impedes LA-producers’ growth. Neutral agents, such as calcium hydroxide (Ca(OH)_2_), sodium hydroxide (NaOH), and ammonia, are needed for LA production, which increases the costs of downstream process [3]. There have been various trials to develop the strains with tolerance to acid pH and improve the capacity to produce LA at high yields and productivity. *Saccharomyces cerevisiae* is not a natural LA-producer. However, it has received increasing attentions for industrial LA production because of its acid tolerance. Acid-tolerant *S. cerevisiae* was engineered to produce LA by expressing heterologous lactate dehydrogenase genes and several key pathway genes, including glycerol-3-phosphate dehydrogenase, cytochrome-C oxidoreductase, etc. A concentration of 142 g/L, with production yield of 0.89 g/g and productivity of 3.55 g/L·h, was obtained in fed-batch fermentation [24]. Although fungi have the merits of acid-tolerance and direct-utilization of renewable biomasses, acetic acid and ethanol are produced along with LA, therefore leaving a negative impact on the fermentation. To date, LA bacteria, including *Lactobacillus* strains and *Bacillus* strains, account for 90% of l-lactic acid large-scale production [1].

*Lactobacillus* strains have a long history of industrial LA production. They have great commercial importance due to high acid tolerance, high yield and high productivity of l-lactic acid. Most *Lactobacillus* strains are mesophiles. The low fermentation temperature not only increases contamination risks, but also hampers the uses of lignocellulosic biomass [23]. Thermotolerant strains may minimize contamination problems during LA production. *L. rhamnosus* is a l-lactic acid producer with a thermotolerant temperature up to 42 °C. Aging paddy rice was used as alternative carbon and nitrogen sources for l-lactic acid production by *L. rhamnosus* DUT1908. In one step simultaneous liquefaction, saccharification and fermentation process, 108 g/L l-lactic acid was obtained with a productivity of 3.4 g/L·h and a yield of 0.89 g/g [25]. By combining metabolic engineering and adaptive evolution, a l-lactic acid producer, *L. paracasei* (NCBIO01-M2-ldhL1-HT), was obtained. *L. paracasei* (NCBIO01-M2-ldhL1-HT) produced 221 g/L l-lactic acid in non-sterilized fermentation [26]. *Bacillus* is a thermophilic LA producer. It can produce l-lactic acid at above 50 °C, which reduces energy consumption and contamination risks during production. A thermophilic *Bacillus* sp. strain 2–6 was used in completely open repeated batch fermentation for producing l-lactic acid. Up to 107 g/L l-lactic acid of optical purity 99.8% was obtained with NaOH as pH regulator [27].

### 2.2. Substrates for l-Lactic Acid Production

Nowadays, microbial production of l-lactic acid is performed with pure sugars. The substrate costs constitute approximately 40~70% of the entire costs. The utilization of renewable, lost-cost, and non-food substrates as an alternative for pure sugars is an economical way for LA production [6,49].

#### 2.2.1. Lignocellulosic Biomass

Lignocellulosic biomass has gained increasing attention due to its abundance, non-food sugar constituents, renewability and cost efficiency. Pre-treatment is essential to lignocellulosic biomasses for releasing fermentable sugars. However, the inhibitor compounds, such as hydroxymethylfurfural, furfural, and phenolic acid, are also released [49]. Furthermore, mixed sugars (xylose, glucose, and arabinose) derived from lignocellulosic biomass cannot be efficiently used by most LA-producers.

*Bacillus* strain is one of the widely reported strains for l-lactic acid production using lignocellulosic biomass (Table 1). Wang et al. [28] isolated a *Bacillus* sp. strain XZL9. It could metabolize glucose and xylose simultaneously into only l-lactic acid by the homofermentative pathway. In microorganisms, xylose is firstly converted into xylulose-5-phosphate (X5P). This metabolite is further metabolized through two pathways: the pentose phosphate pathway (PPP) and the phosphoketolase pathway (PKP). For the PKP, xylose is converted to LA and acetic acid. While in the PPP, X5P is converted into LA through the Embden–Meyerhof pathway (EMP). The theoretical value of xylose conversion is 1.0. Xylose is metabolized through the PPP pathway in *Bacillus* sp. strain XZL9. The concentration of l-lactic acid (75 g/L) was produced from corncob molasses. Pre-treatment of lignocellulosic biomass inevitably produced toxic compounds, such as 2-furfural, that inhibit microbial growth. To fully utilize lignocellulosic feedstocks, considerable interest has been focused on the study of tolerance to inhibitory compounds in lignocellulosic hydrolysates. A novel *Bacillus* sp. strain P38 was isolated from the sludge of a sewage treatment plant by using a high concentration of cellulosic hydrolysate as sole carbon source. It had extraordinary tolerance to 10 g/L 2-furfural. The l-lactic acid concentration of 180 g/L was obtained from corn stover hydrolysate, with a high volumetric productivity of 2.4 g/L·h and a yield of 0.96 g/g total reducing sugars [29].

In addition to *Bacillus* strain, strains of other species have been recently reported to have the ability to produce l-lactic acid from a lignocellulosic biomass. A novel strain, *Lactobacillus paracasei*, which has a tolerance to inhibitors derived from lignocellulosic biomass, was isolated. Deletion of the intrinsic d-lactate dehydrogenase enabled the production of 215 g/L l-lactic acid with glucose as carbon resource. A concentration of 99 g/L L-lactic acid was obtained using non-detoxified wood hydrolysate. Rice straw hydrolysate without detoxification was also tested and 67 g/L l-lactic acid was obtained, with a productivity of 5.27 g/L·h [30]. The mixed culture of *B. coagulans* and *L. rhamnosus* was used to produce l-lactic acid from co-saccharified cassava bagasse. The l-lactic acid concentration and productivity of 113 g/L and 2.74 g/L·h was achieved [31]. Nowadays, cassava bagasse, beechwood hydrolysate, sugarcane bagasse, corn stover, and wood hydrolysate have been used as substrates for l-lactic acid production. Pre-treatment of lignocellulosic feedstocks are critical for high-quality LA production. LA production from lignocellulosic biomass is quite challenging with an associated high pre-treatment costs [3].

#### 2.2.2. Food Waste

Food waste has more potential due to its high carbohydrate content and does not require expensive pre-treatment. Kitchen residues, tea leaves, and vegetable leaves have been reported to be feasible for LA production [32]. A pilot-scale study on l-lactic acid production from food waste was reported by Gao et al. [33]. l-Lactic acid production was carried out under sterilized and non-sterilized conditions. A concentration of 34 g/L l-lactic acid was produced, with a productivity of 0.55 g/L·h. Compared with dl-lactic acid, optically pure LA is far more valuable. It attracts industry interests as a precursor for the promising biodegradable plasmics, PLA. Therefore, researchers try to develop reliable and cost-effective approaches to produce optically pure LA. The challenges with l-lactic acid production from food waste are low l-lactic acid yield due to slow hydrolysis rate, consumption of l-lactic acid by other microorganisms, and decreased economic value due to generation of racemic dl-lactic acid [50]. It has been reported that salt addition could enhance the optical purity of l-lactic acid. The mixed substrate of food waste and waste activated sludge were used for l-lactic acid. The optical pure l-lactic acid with a yield of 30 g/L was obtained at 30 g/L NaCl. The reason for the enhanced optical purity of l-lactic acid is perhaps that the activity of d-lactic acid producing enzymes are sensitive to high concentration of salt. Furthermore, high salt concentration resulted in the changes of microbial community and decreased diversity of indigenous microbiota [34,51].

#### 2.2.3. Starchy Materials

Starchy feedstocks have more potential due to their high carbohydrate content and do not require expensive pre-treatment. An engineered *L. plantarum* NCIMB 8826 strain was constructed by deleting d-lactate dehydrogenase gene and lactate racemase gene. The engineered strain could produce optically pure l-lactic acid from raw starch with a concentration of 50 g/L [35]. Cassava is one of the most efficient crops in terms of carbohydrate production. It is a tropical perennial plant that grows on poor or depleted soils where the yields of other crops are very low. Cassava powder, produced by grinding cassava to powder, was used for l-lactic acid production by Wang et al. [36]. The high l-lactic acid concentration (175 g/L) was obtained in simultaneous saccharification and fermentation. This is the highest l-lactic acid concentration reported, from cassava source. Jerusalem artichoke is a low-requirement crop with high sugar content. l-Lactic acid was produced from the hydrolysates of Jerusalem artichoke powder by a thermophilic bacterium, *B. coagulans* XZL4. High l-lactate production (134 g/L) was obtained using Jerusalem artichoke powder and corn steep powder in fed-batch fermentation, with an average productivity of 2.5 g/L·h and an optical purity of 99.5% [37]. Despite huge potentials and recently growing interest in commercial LA production from starchy biomass, literature lacks studies that evaluate the techno-economic feasibility of its commercial production. Manandhar and Shah [52] estimated the resources required including equipment, chemicals, consumables, utilities, and labor for commercial scale LA production based on three fermentation pathways using either LA producing bacteria, fungi or yeast. Results showed that LA production costs were highly sensitive to sugar-to-LA conversion rates, materials’ price, plant size, annual operation hours, and potential use of gypsum. Improvements in process efficiencies and lower equipment and chemical costs would further reduce the cost of LA production.

## 3. d-Lactic Acid Biosynthesis

Both l-lactic acid and d-lactic acid are the precursors for PLA production. Compared with the extensive investigation of l-lactic acid production, there are relatively few studies on d-lactic acid fermentation. Approximately 70% of LA is used in the food industry because of its role in the production of yogurt and cheese. Because d-lactic acid cannot be metabolized by the human body, studies on d-lactic acid fermentation are limited [2]. Recently, the increasing use of PLA has led to a surge in the demand for d-lactic acid [3]. A few wild-type strains, such as *L. delbrueckii*, *B. laevolacticus*, *L. coryniformis*, *Corynebacterium glutamicum*, and *L. bulgaricus*, have been reported to be homofermentative d-lactic acid producers. Furthermore, metabolically engineered *S. cerevisiae* and *E. coli* have been reported for the production of optical pure d-lactic acid. The titer of d-lactic acid production is generally much lower than that of l-lactic acid [38].

### 3.1. d-Lactic Acid Producing Strains

A highly efficient d-lactic acid producer, *Sporolactobacillus* sp. CASD, was reported to produce 207 g/L d-lactic acid, with the average productivity of 3.8 g/L h and optical purity of 99.3% (Table 1) [38]. To our knowledge, this is the highest d-lactic acid production. These values are comparable to those obtained in l-lactic acid. Thermophilic *B. coagulans* can utilize a broad range of inexpensive carbon resources and produce optically pure l-lactic acid at 50~55 °C, which is expected to minimize contamination during fermentation in industrial scale. To obtaining a thermophilic d-lactic acid producer, an optically pure l-lactic acid producer, *B. coagulans* DSM1, was chosen for genetic engineering. By replacing the key gene for l-lactic acid production with *LdhD* from *L. delbrueckii* subsp. *bulgaricus* DSM 20081, the genetically engineered strain produced high optical purity of d-lactic acid under non-sterilized condition [5]. More recently, *S. cerevisiae* was systematically engineered to produce d-lactic acid by overexpressing d-lactic acid producing genes and deleting glycerol pathway genes. A concentration of 40 g/L d-lactic acid was achieved, with a yield of 0.81 g/g [39]. LA production requires the addition of neutralizing agent in the medium. Acid-tolerant *S. cerevisiae* was constructed by using the CRISPR-Cas-mediated genome evolution method. Approximately 34 g/L d-lactic acid was produced in non-neutralized condition, and 52 g/L d-lactic acid was obtained in a semi-neutralized condition [40].

*C. glutamicum* is a Gram-positive soil bacterium, which has been widely used for the industrial production of amino acid. Its genome is sequenced, and genetic engineering tools are available. *C. glutamicum* was developed to produce LA through intensive metabolic engineering including the introduction of the Entner–Doudoroff (ED) pathway genes, overexpression of glycolytic genes, and modulation of redox balance. Finally, the production of 264 g/L d-lactic acid was obtained, with an optical purity of 99.9% [41]. *E. coli* strains have simple nutritional requirements and are easily genetically manipulated. They are ideal cell factories for the production of metabolic products. Several studies reported the use of engineered *E. coli* strains for LA production from glucose, xylose, sucrose, and glycerol. However, the final concentration (≤63 g/L) and fermentation temperature (~37 °C) by engineered *E. coli* strains were much lower than that achieved with many LA bacteria and *Bacillus* species [23]. The PR-PL promoters were exploited as a genetic switch to regulate the expression of lactate dehydrogenase and the subsequent production of LA in *E. coli*. The d-lactate dehydrogenase promoter, PldhA, was replaced by PR-PL promoters (as a genetic switch), resulting in the thermo-controllable strain B0013-070B. A concentration of 123 g/L d-lactic acid was obtained at 42 °C [42]. Recently, an *E. coli* strain was manipulated for its glycerol dissimilation and d-lactic acid synthesis pathways. Combining adaptive evolution under high crude glycerol, a titer of 115 g/L d-lactic acid was obtained in batch fermentation, with a productivity of 3.29 g/L·h [43].

### 3.2. d-Lactic Acid Production from Renewable Resources

Manufacturing commercially viable d-lactic acid is desirable compared to petrochemical resources. The production of d-lactic acid from corn stover, brown rice, and hardwood pulp hydrolysate has been studied. Orange peel waste was used as raw materials for d-lactic acid production using *L. delbrueckii* ssp. *bulgaricus* CECT 286. The concentration of 45 g/L d-lactic acid was produced, with an optical purity of 99.5% [44]. Dried Distiller’s Grains with Soluble (DDGS) hydrolysate was used as a substrate for d-lactic acid production by *Lactobacillus coryniformis* subsp. *torquens*. Two strategies of separate hydrolysis and fermentation (SHF) and simultaneous saccharification and fermentation (SSF) were used, and a concentration of 38 g/L was obtained in SSF, with an optical purity of 99.9% [45]. Molasses and corn steep liquor were used for d-lactic acid production by *L. delbrueckii*. A high titer of d-lactic acid (162 g/L) was achieved after 48 h of fermentation with a productivity of 3.37 g/L·h [46]. During the utilization of sugarcane molasses and soybean meal, adaptive evolution was used in *L. delbrueckii* S-NL31 in order to improve d-lactic acid concentration. Finally, fed-batch simultaneous enzymatic hydrolysis of soybean meal and fermentation process by evolved strain resulted in d-lactic acid levels of 112 g/L, with an average production efficiency of 2.4 g/L·h and optical purity of 99.6% [47]. In another study, the co-culture batch process of *L. delbrueckii* and engineered *L. lactis* was carried out to efficiently produce d-lactic acid from lactose or whey-derived lactose. d-Lactate dehydrogenase gene together with galactose permease gene was over-expressed in *L. lactis*. The recombinant *L. lactis* could convert galactose into d-lactic acid. By co-culturing *L. delbrueckii* and engineered *L. lactis*, approximately 45 g/L d-lactic acid was achieved from whey permeate [48]. l-Lactic acid production has been extensively studied using several strains and substrates including lignocellulosic hydrolysates, food wastes and starchy materials. Some companies, such as Corbion (Amsterdam, The Netherlands), Galactic (Celles, Belgium), and NatureWorks LLC (Minnetonka, MN, USA), are operating on l-lactic acid production using renewable resources [53]. d-Lactic acid production using renewable resources is quite limited. Due to the significance in the production of PLA, d-lactic acid production from renewable resources is currently in the spotlight. There is still need for research of efficient d-lactic acid production process, especially in terms of utilization of cheap resources.

## 4. Strategies for Clean Fermentation Technology of Lactic Acid

The downstream recovery of LA is one of the biggest challenges in LA production. The cost of LA purification accounts for approximately 50% of the total cost. Moreover, a large amount of solid waste (calcium sulfate) is produced during the operation, which makes them environmentally unfriendly. Therefore, it is necessary to search for other economical, efficient and environmentally friendly techniques for LA production [49]. The utilization of sodium hydroxide (NaOH) instead of calcium carbonate (CaCO_3_) as a neutralizer of LA fermentation can solve the problem of environmental pollution caused by the traditional addition of CaCO_3_ [1]. Alkaliphiles grow optimally at a pH above 9. They are also tolerant to salt, especially those of monovalent ions, such as sodium ions. Alkaliphilic strains may be promising producers of organic acids and their tolerance to high levels of salt and a high pH could also minimize contamination. An alkaliphilic strain *Bacillus* sp. WL-S20 was isolated from a marine environment. In multi-pulse fed-batch fermentation, a l-lactic acid concentration of 225 g/L with a yield of 99.3% was obtained [54]. The high concentration of optically pure l-lactic acid produced by an alkaliphilic strain using environment-friendly NaOH-based process provides a potentially novel way for LA production at an industrial scale. Another alkaliphilic strain, Enterococcus hirae BoM 1–2, was isolated to production LA using NaOH as neutralizer. The LA concentration of 181 g/L was achieved in a multi-pulse fed batch strategy with volumetric productivity of 0.65 g/L·h [55]. Alkalophilic microorganisms are the crucial sources for LA fermentation. The concentration of LA produced by alkaliphilic strains was higher than that produced by the non-alkaliphilic counterparts. Alkalophilic microorganisms enriched the LA fermentation strain resources and provided a new idea for the development of new LA fermentation and separation coupling fermentation process [56].

Except for alkaliphilic strains, membrane-based hybrid reactor systems could also be used for clean production of lactic acid. Membrane based hybrid reactor system successfully stands in that objective without creating any negative environmental impacts. More than 95% removals of impurities were achieved in the hybrid reactor system, with a purity of 95% [57]. Membrane separation technology has the advantages of high separation efficiency, mild operating conditions, non-toxic separation medium, environmentally friendly, and reliable process amplification [1]. A two-step electrodialysis system was established to purify LA. Firstly, the fermentation broth was clarified by microfiltration membrane to remove bacteria and macromolecular proteins. Due to the calcium salt regulation process adopted in the fermentation, the divalent metal ions in the fermentation broth are easy to cause membrane pollution in the process of bipolar membrane operation. The fermentation broth is again removed by the nanofiltration process. The clarified fermentation broth is concentrated by electrodialysis in the first step, and then transformed into LA and corresponding alkali by secondary electrodialysis of bipolar membrane. The alkali is returned to the fermentation tank and used as neutralizer again [58]. The use of NaOH as neutralizer can avoid the pollution of divalent metal ions to the membrane. Therefore, the development of new LA producing strains, such as alkaliphilic strains, can significantly improve the economy of membrane separation.

## 5. Polylactic Acid (PLA) Biosynthesis

Relative to LA, the most of which have been produced by microbial fermentation, PLA are mainly synthesized by the chemical polymerization process of LA (ROP of lactide) to date. Recently, the direct one-step fermentative processes for the production of PLA and several LA-containing polyesters have been developed by employing metabolically engineered microorganisms [59]. In this biosynthesis process, the most critical is to develop two key catalytic enzymes, propionyl-CoA transferase and PHA synthase. Firstly, LA is converted into lactyl-CoA by propionyl-CoA transferase, and then lactyl-CoA is polymerized by PHA synthase (Figure 2b).

### 5.1. Development of Enzyme for Converting LA into Lactyl-CoA

Although there is no enzyme that specifically catalyzes LA to produce lactyl-CoA in nature, previous reports have shown that propionyl-CoA transferase (Pct), found from several microorganisms including *C. propionicum*, *Megasphaera elsdenii*, *Bacteroides ruminicola*, and *C. homopropionicum* in alanine fermentation pathway, can transfer CoA from propionyl-CoA or acetyl-CoA to LA to form lactyl-CoA [16,17,60]. However, the two propionyl-CoA transferases from *C. propionicum* (Pct*_Cp_*) and *M. elsdenii* (Pct*_Me_*) were also found to strongly inhibit the growth of the cell when expressed in *E. coli*. To solve this issue, random mutagenesis was performed to create several Pct*_Cp_* mutants harboring the enhanced ability to supply lactyl-CoA in *E. coli* without severe growth inhibition. Among these positive Pct*_Cp_* mutants, two beneficial Pct*_Cp_* mutants, Pct532*_Cp_* (A243T, and one silent nucleotide mutation of A1200G) and Pct540*_Cp_* (V193A, and four silent nucleotide mutations of T78C, T669C, A1125G, and T1158C), which led to an increase in both the polymer content and lactate mole fraction in the PLA copolymer [17]. Although, the positive Pct*_Cp_* mutants had no inhibition on cell growth, the catalytic activity of the enzyme still cannot meet the requirements of efficient synthesis of lactyl-CoA. Recently, it has been found that CoA transfers from *Roseburia* sp., *Eubacterium hallii*, *Faecalibacterium prausnitzii*, and *Anaerostipes caccae* can also convert LA into LA-CoA [61]. Furthermore, isocaprenoyl-CoA:2-hydroxyisocaproate (2HIC) CoA-transferase (HadA) from *Clostridium difficile* was also found to be capable of activating LA into lactyl-CoA in addition to its original substrate, 2HIC [62,63]. All these CoA transferases above can activate LA to produce LA-CoA, which provides abundant enzyme resources for the biosynthesis of PLA. Some of the CoA-transferases capable of production of lactyl-CoA are summarized in Table 2.

### 5.2. Development of Enzyme for Polymerization of Lactyl-CoA into PLA

Apart from CoA-transferase, another key enzyme involved in PLA synthesis is PHA synthase, which generally used as the main enzyme of polyhydroxyalkanoates (PHAs) biosynthesis. Depending on the carbon numbers PHAs are classified into major two groups with different material properties: short-chain-length (SCL) and medium-chain-length (MCL)-PHAs. SCL-PHAs are composed of monomers having 3 to 5 carbon atoms and display thermoplastic material properties such as polypropylene. MCL-PHAs are composed of monomers with 6 to 14 carbon atoms and have elastic material properties similar to rubber and elastomer. In microorganisms, many bacteria naturally accumulate PHAs in their cytoplasm as carbon and energy storage materials when they encounter limited growth conditions in the presence of excess carbon sources [61]. However, the natural PHA synthases generally accept 3-hydroxyacyl-CoAs as the most favorable substrates, and 4-, 5- and 6-hydroxyacyl-CoAs can also be used as substrates but showed no or only slight activities on lactyl-CoA [15,72].

Depending on the subunit compositions and substrate specificities of the PHA enzymes, they are generally classified into four groups: class I, II, III, and IV (Table 3) [73]. Class I and II PHA synthases are composed of a single one subunit enzyme, PhaC. Class I PHA synthases such as *Ralstonia eutropha* and *Alcaligenes latus* PHA synthase accept short-chain-length-HA-CoAs for polymerization [74], while class II PHA synthases mainly from *Pseudomonads* display substrate specificity towards medium-chain-length-HA-CoAs [75]. Some class II PHA synthases from *Pseudomonas* sp. 61-3 [76] accept both SCL- and MCL-monomers, with much weak activity towards SCL-monomers. Class III PHA synthases are composed of two different subunits, PhaC and PhaE [77]. These subunits have much low sequence homology to class I and II PHA synthases; for example, the PhaC subunits display only 20–30% homology with each other. Class III PHA synthases are highly specific for SCL-HA-CoAs, but also accept MCL-HA-CoAs as substrates when expressed in some *Pseudomonads* [78]. Class IV PHA synthases are composed of two different subunits, PhaC and PhaR, which are usually found in *Bacillus* strains producing P(3HB) [79,80]. All the PHA synthases except for *Pseudomonas* sp. MBEL 6–19 PhaC1 showed poor activity to the substrate.

In order to obtain the PHA synthase that efficiently catalyzes lactyl-CoA, PHA synthases from *Pseudomonas* MBEL sp. 6-19 and *Pseudomonas* sp. 61-3 were selected to perform site-directed mutagenesis and resulting variants that had amino acid residues substitutions of Glu130Asp, Ser325Thr, Ser477Arg/His/Phe/Gly, as well as Gln481Lys/Met were effective for in vivo catalysis of lactyl-CoA [16,17,64]. Similarly, other PHA synthases from different *Pseudomonas* strains were also engineered through site-directed mutagenesis and the resulting variants showed enhanced substrate specificity toward lactyl-CoA [81].

### 5.3. Metabolic Engineering for Production of PLA

By employing the two key enzymes of CoA transferase and engineered PHA synthase, the microorganisms assembled with both LA biosynthetic and LA polymerizing pathways have been further developed for the cell factory, which efficiently converts the cheap carbohydrates such as glucose into PLA in vivo. PLA firstly was produced in recombinant *E. coli* with a significantly low content, 0.5 wt%, of dry cell weight [17]. Even though such a system is suitable for a proof-of-concept study, it is not preferred for the industrial-level production of polymers. Accordingly, many studies focused on the engineering metabolic pathways of host strains to provide more precursors LA [82,83].

With metabolic pathways engineered by knocking out the *ackA*, *ppc*, and *adhE* genes encoding acetic acid kinase, phosphoenolpyruvate carboxylase and aldehyde dehydrogenase, respectively, and by replacing the promoters of the *ldhA* and *acs* genes with the strong *trc* promoter, the resulting strain *E. coli* JLX10 equipped with evolved class II PhaC1 from *Pseudomonas* sp. MBEL 61-9 and *C. propionicum* Pct can produce P(14mol%3HB-co-86 mol%LA) with high LA fraction, and PLA could be produced up to 11 wt% of dry cell weight when 20g/L glucose supplied [84]. Recently, except for *E. coli*, *Sinorhizobium meliloti* as the native polymer producer also can produce PLA up to 3.2 wt% dried cell weight when expression of *C. propionicum* propionate CoA transferase (Pct532Cp) and an evolved *Pseudomonas* sp. MBEL 6-19 PHA synthase 1 (PhaC1Ps6-19, PhaC1400).

So far, even though PHA containing high fraction of lactate and even PLA homopolymer has been produced employing recombinant *E. coli* expressing evolved PHA synthase, it is almost impossible to generate a truly 100% PLA homopolymer because PHA synthase evolved to accept lactyl-CoA as substrate still has significantly greater substrate specificity towards 3HB-CoA than lactyl-CoA [85]. Conversely, it seems to be possible to obtain PLA homopolymer if the monomer molecule for PLA synthesis such as 3HB-CoA is present in a quantity that is too low to be detected [73]. Therefore, future research should focus more on improving the substrate specificity of PHA synthase and increasing the yield of PLA.

### 5.4. Metabolic Engineering for Production of LA-Containing Copolymers

As mentioned above, PHA synthase is well known for its broad substrate availability towards various hydroxycarboxylic acids (HAs). Therefore, it is relatively easy to incorporating several other HAs monomers with LA to produce various LA-containing copolymers by employing the engineered PHA synthase. Originally, LA-containing copolymers generally consisted of natural monomers, including 3-hydroxybutyrate (3HB) [16,84], 3-hydroxypropionate (3HP) [81] and 4-hydroxybutyrate (4HB) [65]. Recently, some novel LA-containing copolymers have been synthetized by employing monomers of 2-hydroxybutyrate (2HB), 2-hydroxyisovalerate (2HIV), 2-hydroxyisocaproate (2HIC) and 2-hydroxy-3-methylvalerate (2H3MV) [62], phenyllactate (PhLA), mandelate (MA) and 4-hydroxyphenyllactate (4HPhLA). Poly(lactate-co-glycolate) and poly(lactate-co-glycolate-co-2-hydroxybutyrate) were produced from xylose as a sole carbon source by using five different synthetic promoters for the expression of *Caulobacter crescentus* XylBC in *E. coli* [86]. P(2HIV-co-2HB-co-3HB-co-LA) was produced in *E. coli* by metabolic engineering including overexpression of feedback resistant *ilvBN* mut genes encoding acetohydroxyacid synthase and *ilvCD* genes encoding ketol-acid reductoisomerase and dihydroxyacid dehydratase, respectively, and *panE* gene encoding (D)-2-hydroxyacid dehydrogenase, and *pct540* gene encoding evolved propionyl-CoA transferase and phaC1437 gene encoding evolved PHA synthase were also overexpressed, along with *ilvBN* mut, *ilvCD*, and *panE* genes [87]. Additionally, the aromatic polyesters poly(3HB-co-D-phenyllactate) can be produced from glucose as a sole carbon source by additional expression of *Ralstonia eutropha* ketothiolase (*phaA*) and reductase (*phaB*) genes [63]. As another novel biopolymer, the quaterpolymer P(3HB-co-LA-co-3HHx-co-3HO) was produced in *P. putida* with polymer content of 42% dry cell weight when cultured in defined media with the addition of sodium octanoate [20].

## 6. Concluding Remarks and Outlook

LA is a versatile green platform compound. LA and its derivatives have been widely used in food, medicine, environmental protection, biodegradable polymer production and other industrial fields. The demand for LA and its derivatives is increasing. With the increasing cost of glucose and other raw materials, the production cost of LA is also increasing, which has seriously affected the profit margin of the LA industry. The economic and technical analysis showed that the concentration of LA should be higher than 180 g/L and the conversion rate could exceed 95% at an industrial scale in the future [1]. At present, the reported strains showed low utilization efficiency for cheap raw materials. It is necessary to optimize the utilization rate of substrates and improve the industrial adaptability of strains through biological engineering. Furthermore, the development for extremophilic LA producers, including thermophiles, acidophiles, and alkaliphiles, can minimize contamination problems during processing. This will be a direction for low-cost LA fermentation.

Although it is now possible to produce PLA by the one-step fermentation of engineered microorganisms, this complete biosynthesis process still faces many challenges. Firstly, the PLA yield and productivity is still low; the contents were below 11 wt%, and the titer and productivity were about 0.6 g/L and 0.02 g/L·h, respectively, which cannot reach the requirements of industrial application and has totally no price advantage compared with chemical synthesis. Thus, the production capability of this microbial PLA synthesis system needs to be much improved for future commercialization. For instance, to enhance the conversion of pyruvate to lactic acid by designing a novel multi-substrate co-utilization pathway will release the inhibition of acetic acid on host strains and improve the production capability of PLA, according to reported strategy [88]. Besides, the average molecular weight of PLA synthesized by engineered PHA synthases was found to be lower than 50,000 Da, which is not acceptable for many polymer applications. It has been suggested that the expression level of PHA synthase is one of the major factors determining the molecular weight of PHA [89]. Thus, PHA synthase needs to be further engineered to accept lactyl-CoA more efficiently and consequently to increase the molecular weights of PLA and lactate-containing polyesters. Considering many successful cases of microbial production systems that have greatly increased compound production through employing systematic metabolic engineering strategies involved in synthetic biology, protein engineering, and evolutionary engineering, it is expected that the microorganism can be a versatile and powerful platform for production of PLA, LA-copolymers, and other non-natural polymers.

## Figures and Tables

**Table 1:** Lactic acid production in microorganisms.

Microorganisms	Substrates	Fermentation Mode	Yield (g/L)	Productivity (g/L·h)	Reference
l-lactic acid producers					
*Rhizopus oryzae*	Glucose	One-step fermentation with fed-batch strategy	162	6.23	[22]
*Saccharomyces cerevisiae*	Glucose	fed-batch fermentation	142	3.55	[24]
*Lactobacillus rhamnosus*	Starchy biomass	One step simultaneous liquefaction, saccharification and fermentation	108	3.40	[25]
*Lactobacillus paracasei*	Glucose	Non-sterilized fermentation	221	7.50	[26]
*Bacillus* sp. 2–6	Glucose	Non-sterilized repeated batch fermentation	107	3.06	[27]
*Bacillus* sp. strain XZL9	Corncob molasses	Fed-batch fermentation	75	0.38	[28]
*Bacillus* sp. strain P38	Cellulosic hydrolysate	Fed-batch fermentation	180	2.40	[29]
*Lactobacillus paracasei*	Non-detoxified wood hydrolysate	Fed-batch fermentation	99	2.25~3.23	[30]
*Lactobacillus paracasei*	Rice straw hydrolysate	Batch fermentation	67	5.27	[30]
*Bacillus coagulans*+ *Lactobacillus rhamnosus*	Cassava bagasse	Simultaneous saccharification and co-fermentation	113	2.74	[31]
*Bacillus coagulans*	Bakery waste and lucerne green juice	Batch fermentation	62	2.59	[32]
*Lactobacillus paracasei* subsp. *paracasei*2	Food waste	Batch fermentation	34	0.55	[33]
Indigenous microbiota	Food waste and waste activated sludge	Batch fermentation	30	0.63	[34]
*Lactobacillus plantarum*	Raw corn starch	Batch fermentation	50	—	[35]
*Lactobacillus rhamnosus*	Cassava powder	Simultaneous saccharification and fermentation	175	3.40	[36]
*Bacillus coagulans*	Jerusalem artichoke powder	Fed-batch fermentation	134	2.50	[37]
d-lactic acid producers					
*Bacillus coagulans*	Glucose	Fed-batch fermentation	145	1.50	[5]
*Sporolactobacillus sp. CASD*	Glucose	Fed-batch fermentation	207	3.80	[38]
*Saccharomyces cerevisiae*	Glucose	Fed-batch fermentation	40	0.83	[39]
*Saccharomyces cerevisiae*	Glucose	Semi-neutralizing fermentation	52	2.17	[40]
*Corynebacterium glutamicum*	Glucose	Fed-batch fermentation	264	3.30	[41]
*Escherichia coli*	Glucose	Shake flask experiment	123	4.39	[42]
*Escherichia coli*	Glycerol	Batch fermentation	115	3.29	[43]
*Lactobacillus delbrueckii* ssp. *bulgaricus*	Orange peel waste	Separate hydrolysis and fermentation	45	0.63	[44]
*Lactobacillus coryniformis* subsp. *torquens*	Dried distiller’s grains with solubles hydrolysate	Simultaneous saccharification and fermentation	38	0.80	[45]
*Lactobacillus delbrueckii*	Molasses and corn steep liquor	Fed-batch fermentation	162	3.37	[46]
*Lactobacillus delbrueckii*	Sugarcane molasses and soybean meal	Fed-batch fermentation	112	2.40	[47]
*Lactobacillus delbrueckii + engineered Lactococcus lactis*	Whey permeate	Fed-batch co-culture process	~45	0.63	[48]

**Table 2:** Some of the CoA-transferases capable of production of lactyl-CoA.

Enzyme Type	Source	Reference
Butyryl-CoA transferase (Bct)	*Roseburia* sp.	[61]
	*Eubacterium hallii*	
	*Faecalibacterium prausnitzii*	
	*Anaerostipes caccae*	
Isocaprenoyl-CoA:2HIC CoA-transferase (HadA)	*Clostridium difficile*	[62,63]
Propionyl-CoA transferase (Pct)	*Clostridium propionicum*	[17,64,65,66,67]
	*Megasphaera elsdenii*	[16,68]
	*Clostridium perfringens*	[69]
	*Cupriavidus necator H16*	[70,71]

**Table 3:** The classification of PHA synthases.

Class	Subunit Composition	Species	Substrate
I	PhaC+ PhaC	*Ralstonia eutropha*	C_3_–C_5_
II	PhaC+ PhaC	*Pseudomonads* sp.	≤C_6_(or C_4_)
III	PhaC+ PhaE	*Allochromatium vinosum*	C_3_–C_6_
IV	PhaC+ PhaR	*Bacillus* sp.	C_3_–C_5_
